# Sexual quality of life in young gynaecological cancer survivors: a qualitative study

**DOI:** 10.1007/s11136-023-03386-1

**Published:** 2023-03-22

**Authors:** Melanie Roussin, John Lowe, Anita Hamilton, Lisa Martin

**Affiliations:** School of Health and Behavioural Sciences, University of the Sunshine Coast, 90 Sippy Downs Drive, Sunshine Coast, QLD 4556 Australia

**Keywords:** Psychosexual distress, Sexual function, Ovarian cancer, Cervical cancer, Intimacy, Gynecologic oncology

## Abstract

**Purpose:**

The impact of cancer diagnosis and treatment on sexual quality of life (SQoL) is a significant and often neglected issue in the treatment and survivorship period of young gynaecological cancer survivors (YGCS). This study sought to explore women’s lived experiences to understand how to protect and improve SQoL.

**Methods:**

A qualitative study with women aged 18–45 and pre- or perimenopausal at diagnosis (*n* = 15). A thematic analysis was performed in NVivo. Participants also completed a pre-interview questionnaire and *The Female Sexual Distress Scale-Revised (FSDS-R).*

**Results:**

YGCS experienced high psychosexual distress. Notably, seven themes were identified: adjustment, confidence, fear, loss, shame, trauma, and communication. Gynaecological cancer (GC) treatment interfered with everyday life and had a long-term impact on mental, physical, and emotional health, with many reporting an altered sense of self, body image and sexual identity. Single women felt vulnerable in new relationships, while partnered women reported low sexual desire and guilt about sexual difficulties. Open communication, emotional intimacy, and an acceptance of the ‘new normal’ buffered the trauma of cancer and were vital to relationship satisfaction. Lastly, absent, or blunt patient-clinician communication contributed to psychosexual distress.

**Conclusion:**

GC interferes with sexual function, partner relationships, psychosexual wellbeing, and quality of life. A better understanding of the lived experiences of YGCS can help healthcare providers to adopt a holistic, patient-centric, and multidisciplinary approach to SQoL. YGCS want psychosexual communication and support, across all stages of treatment and care. Healthcare providers should initiate and normalise conversations on the impact of treatment on SQoL.

**Supplementary Information:**

The online version contains supplementary material available at 10.1007/s11136-023-03386-1.

## Plain English summary

The impact of cancer diagnosis and treatment on sexual quality of life (SQoL) is an important issue in the treatment and survivorship period of young gynaecological cancer survivors (YGCS). Still, sexuality is often not addressed by the healthcare system, despite affecting the quality of life (QoL) of younger women many years after treatment. In this study, we explored the experiences of YGCS to understand how to protect and improve SQoL. This study indicates that gynaecological cancer (GC) interferes with sexual function, partner relationships, psychological wellbeing, and QoL and that YGCS want communication and support for long-term psychosexual care throughout their cancer journey. Healthcare providers should initiate and normalise conversations on the impact of treatment on SQoL and work together to offer solutions based on patient needs. Findings from this study provide new insights for healthcare providers on the impact of cancer and its treatment on SQoL for YGCS, inviting them to become more engaged in the conversation around SQoL. Finally, this study inspires more research on the SQoL of YGCS through the lens of trauma-informed care.

## Introduction

Severe psychosexual distress is widely reported among gynaecological cancer patients and survivors (GCS), often negatively impacting quality of life (QoL) [1–3]. Gynaecological cancer (GC) treatment is associated with physical and psychological complications including hormonal and vaginal changes (e.g. scarring, shortening, dryness, discharge), early menopause, infertility, lymphoedema, pelvic floor disorders, pain, fatigue, anxiety, urinary and faecal incontinence, body image concerns, and feminine identity crisis [4–8]. These invasive and often persistent symptoms are linked to sexual difficulties such as a changed sexual self-concept, fear of sex, painful sex, infrequent sexual activity, and lack of sexual arousal, desire, and orgasm [4, 9–12].

A systematic review of the factors of SQoL in GC [13] revealed that age and menopausal status are risk factors to SQoL. Issues such as sexual dysfunction and impaired libido may affect both pre- and post-menopausal GCS [4, 14, 15]. Premenopausal individuals may be more vulnerable to experiencing anxiety surrounding infertility, loss of womanhood or fear of rejection by their partners [9, 10]. Evidence also shows that menopause can alter sexual response [9] and that experiencing premature menopause can lead to psychological distress and lower QoL [5, 7]. Moreover, younger survivors have reported feeling alone [16], dissatisfied in their intimate relationships [9], and worried about body image and sexual concerns [17, 18]. Lastly, Komblith et al. [18] found that YGCS experienced significantly worse adaptation on a range of QoL measures related to psychological wellbeing, cancer treatment and sexual problems. Despite these findings, the literature dedicated to YGCS and SQoL remains scarce. Understanding the lived experiences and perspectives of YGCS about psychosexual wellbeing, cancer treatment, partner relationships, and the healthcare system may help reduce the burden of treatment sequalae. This study sought to understand how we can protect and improve SQoL using a holistic and salutogenic approach to sexuality [9, 13, 19].

## Methods

### Design

We performed a qualitative study of women living in Australia and who were aged 18–45 and pre- or perimenopausal at their GC diagnosis. We conducted individual interviews on a video conferencing platform, Zoom. Semi-structured interviews allowed for an in-depth exploration of participants’ lived experiences and perspectives on SQoL. Three pilot interviews with YGCS ensured clarity of the questions and robustness of the interview design. Data from the pilot interviews was not included in the final data set but helped to a) streamline the interview schedule, b) ensure participants felt comfortable discussing sensitive topics on Zoom, and c) confirm that scheduling 60–90 min was sufficient. Participants also completed pre-interview questionnaires. The research received ethical approval by USC Human Research Ethics Committee (S201448).

### Recruitment

The approach to recruitment capitalised on leveraging ‘influencers’ (individuals well known in the YGC community), charities and social media communities providing GC education and support. A grassroots campaign using a combination of social media posts, direct messaging, email and website mentions was implemented to recruit participants with an emphasis on organic reach and content shareability [20]. Achievement of data saturation guided the final number of participants interviewed.

### Eligibility

Participants’ consent and eligibility were registered via an online questionnaire. Eligible participants were 1) diagnosed with GC between 18–45 years old 2) pre- or perimenopausal at diagnosis (as defined by the presence of at least 2 menstrual periods within 6 months of diagnosis), 3) residing in Australia, and 4) willing to discuss their experience on sexuality and cancer. Women were excluded if they had insufficient English, no access to a smart phone or computer with a reliable Internet connection, recurrence of GC or a diagnosis of another cancer or medical condition that could have impacted their ability to participate. The study was open to single and partnered women.

### Data collection

Participants were given information in the *Research Project Information Sheet* about what would be included in the interviews. Upon confirming their consent via a secure online platform, a unique 4-digit code was randomly assigned to protect anonymity and to match data across time. Participants completed three short questionnaires. The first questionnaire consisted of nine screening questions to confirm eligibility. The second questionnaire was the *Female Sexual Distress Scale-Revised (FSDS-R)*, a 13-item screening questionnaire used in other studies with GCS [21, 22]. The third questionnaire aimed to create an overall profile of the participants and to identify any characteristics that may benefit future research. Interviews were arranged via email and lasted 60–90 min. The interview guide covered four topics informed by research [13]: (1) the impact of cancer and is treatment on SQoL, (2) relationship satisfaction and emotional support, (3) the role of the healthcare system, and (4) the adaptation to a new normal.

### Data analysis

Questionnaire data was collected and analysed as descriptive statistics using an online data capture tool, Qualtrics [23], and then imported into Excel. All interviews were transcribed verbatim. A thematic analysis [24] of interview transcripts was completed using a qualitative data analysis software, NVivo Version 1.5 [25]. Our analysis followed the steps suggested by Braun and Clarke [26]. According to the authors, thematic analysis is used to tell a story into patterns of meaning (themes) across a data set to answer a research question. Thematic analysis was chosen as a data analysis method for its flexibility in “giving voice” to the lived experiences of YGCS [26]. Thus, our approach was mainly inductive as we coded from the data based on participants’ experiences to uncover how GC impact the SQoL of YGCS. To a lesser extent, our approach was also deductive as we drew on theoretical constructs from the Salutogenic Theory [19] to unveil meaning that relates to all aspects of the person. For quality control, the first author, in consultation with the co-authors with expertise in different areas, followed a two-stage review process [27]. Themes were first organised in categories from data collection questions (initial codes). Then, as we became more familiar with the data, themes were reviewed against the coded data and rearranged into new themes (code clusters) that tell the story as it aligns with our research question [28]. Lastly, the Critical Appraisal Skills Programme (CASP) checklist for Qualitative studies [29] was used as standardised tool to critically appraise the process undertaken.

## Results

### Participant characteristics

Fifteen participants joined the study from across Australia (Table 1), with almost an even split between cervical (*n* = 8) and ovarian (*n* = 7) cancer. All FIGO stages (i.e., cancer stages determined by the International Federation of Gynecology and Obstetrics) [30] and 18–45 age groups were represented. Six participants reported a change in partner or relationship status since diagnosis. One participant was in a same-sex relationship. Cancer treatment varied between participants, including surgery, chemotherapy, radiotherapy, and hormone therapy, with the most common treatment hysterectomy. Nearly two thirds of participants had completed treatment (64%). Almost all questionnaire respondents reported being sexually active (93%), however results from the FSDS-R and interviews suggest low sexual activity (defined as sexual relations and self-pleasure) and severe psychosexual distress. Forty percent of women said they never tried vaginal dilation, but many wished they had known it was an option.

**Table 1:** Participants’ characteristics

Characteristics	Results
Place of residence in Australia	QLD, NSW, ACT, VIC, SA, WA
Cancer site	Cervical cancer (8), ovarian cancer (7)
Cancer stage	Stage I (5), Stage II (5); Stage III (2), Stage IV (3)
Age at diagnosis	18–24 (2), 25–31 (8), 32–38 (4), 39–45 (1)
Relationship status—at diagnosis	Married/de-facto (13), single (2)
Relationship status—current	Married/de-facto (11), dating (3), other (1)
Year diagnosed	Recent and long-term (2005–2021)
Sexual activity	14 participants reported being sexually active
Vaginal dilation	Never (6), occasionally, (5), bi-weekly (1), monthly (1), fortnightly (0), weekly (2)

### FSDS-R results

**Fig. 1:**
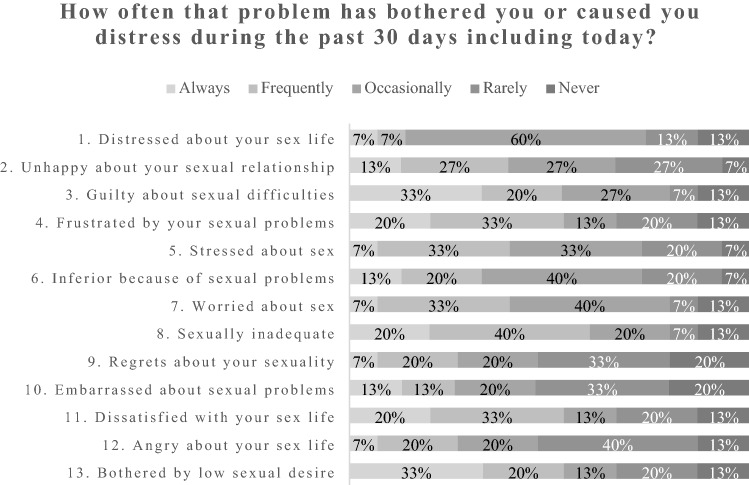
Results of FSDS-R questionnaire

Figure 1 shows that most participants were experiencing psychosexual distress (a total score of greater than or equal to 11 indicates sexual distress). All participants, except two, reported signs of sexual distress. The most common concerns were feeling guilty about sexual difficulties and being bothered by low sexual desire.

### Thematic map

**Fig. 2:**
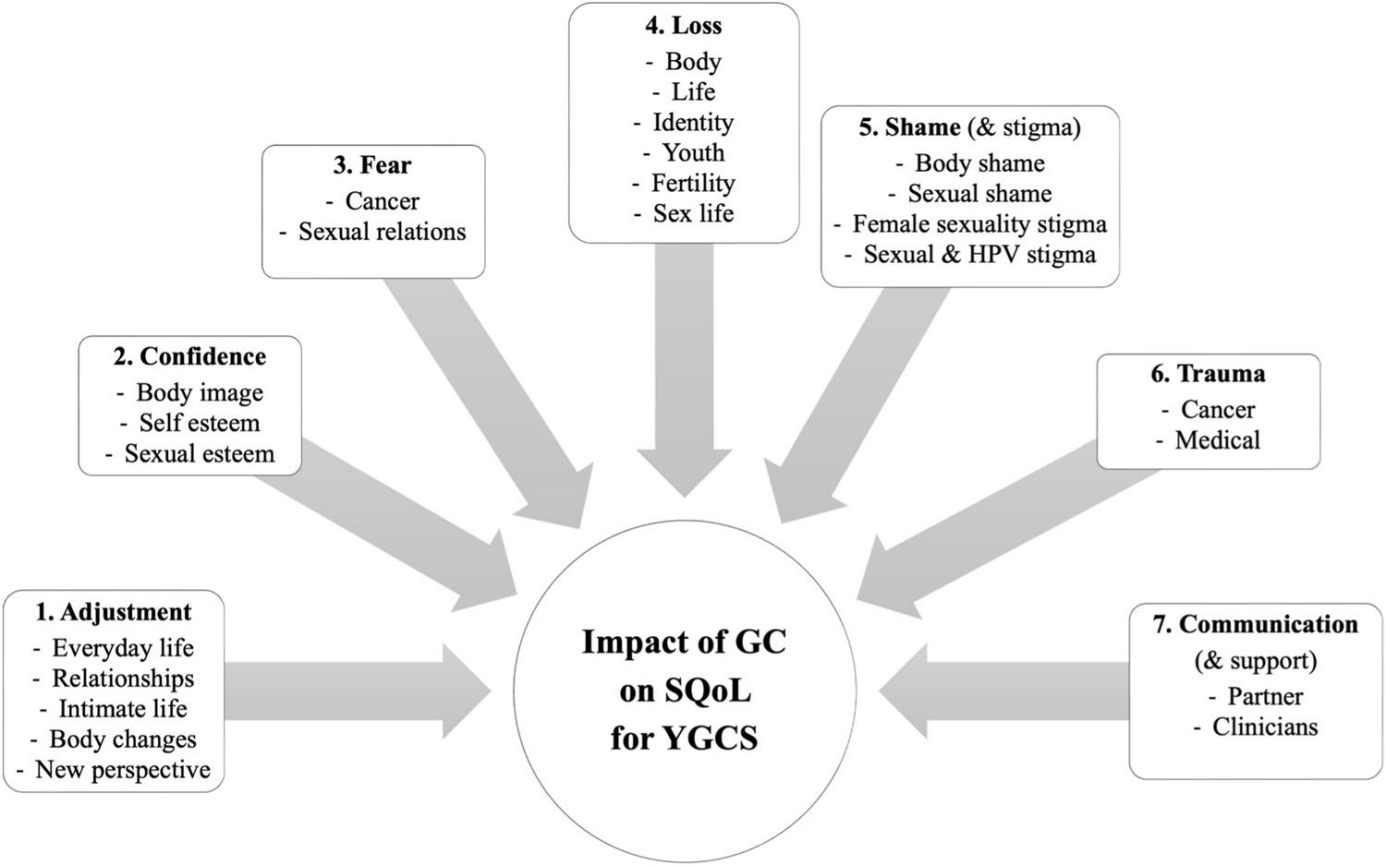
Thematic map

Seven main themes were identified in this analysis: adjustment, confidence, fear, loss, shame, trauma, and communication. Figure 2 shows a thematic map.

### Theme 1: adjustment

Adjustment to gynaecological cancer was reported as an ongoing process of solving cancer-related problems in everyday life, managing body changes, and navigating new dynamics in relationships.

#### Everyday life and relationships

The ripple effects of cancer diagnosis and treatment extended to all aspects of how YGCS live, learn, play, work, and connect: “My capacity to do anything has been greatly reduced. From everything every day, as cooking and cleaning, to working. Even in terms of self-care and enjoyment” (#6260) and “My ability to learn, because my brain is just not what it was” (#2318) as well as “It’s affected all my relationships” (#3611). Specifically, all participants expressed that cancer impacted their partner relationship—whether it was the dissolution of a marriage, some relationship adjustments, or the challenges of dating after cancer.

I'm trying to date and manage the ongoing treatment impact. How do you have those conversations? That has been very challenging, because I'm an introverted person. I like to be capable and having those conversations is almost like saying, ‘There's something wrong with me.’ I don't like to be vulnerable, especially with new partners. The dating world can be quite vicious at the best of times. (#2967)

#### Intimate life and body changes

Cancer treatment can sometimes lead to sudden changes in menopausal status, “When I went to my surgery, there was nothing, then just slammed into menopause. That was a massive adjustment” (#2318). Participants also reported changes in body functions including bladder, bowel, and cognitive function, also referred to as “chemo brain” (#3469). Table 2 shows illustrative quotes directly related to sexual function.

**Table 2:** Sexual function subthemes and illustrative quotes

Subtheme	Illustrative quote
Painful sex	“I suppose we tried but it was too painful. It wasn’t an option.” (#3611)
Impaired sexual desire	“My interest in sex is just non-existent. I have no sex drive.” (#5137)
Changed sexual response	“It's not as wet anymore. My vagina doesn't get as wet.” (#3711)

Furthermore, YGCS reported the adjustment in their intimate life as an unexpected side effect of treatment.

It's really removed my ability to have sex the way that I used to. To the point where I am having to learn a new way of being intimate with my partner, which is a big deal in itself. It's also not something that was ever communicated to me, that would be part of my treatment or this process. (#6260)

#### New perspective

Cancer can lead to a shift in priorities to nurture health and relationships, “For a six-year period, I was very committed to making sure that I didn't get it again. I was very focused on my health and making new habits. My children were my focus.” (#2788) Moreover, when asked: “has cancer had any positive effects on you?”, a common response was: “A new perspective on life. An understanding of things that are actually important. Not sweating the small things” (#1634). A sense of gratefulness was also expressed, “I’m very grateful, I found my purpose through it” (#2788) or “I feel I have the permission now to live life to my standards of happiness” (#6260). Finally, some women said their cancer journey led to new connections, “I’ve met some amazing people that I wouldn’t have met otherwise” (#2318) and sometimes becoming more “empathetic with friends” (#5166).

### Theme 2: confidence

#### Body image

Confidence can be shaken by body changes from cancer treatment, such as hair loss, scars, and weight changes, “I really hate the way I look now. My weight is a huge issue” (#2698). For YGCS, this can be expressed as a mix of frustration and pride for what their bodies have endured.

Even though my body has failed me, in a sense of what the surgery has done, I was so proud of my scar. I was like, ‘Look at what I went through’. I counted the staples, because I wanted to know if I could say, ‘I have 50 staples.’ (#2318).

Younger women also described a cycle of re-embodiment and disembodiment, “That's probably split in two. One minute, I'm amazed at my body. I've got through this. I feel proud and strong of my body. But also, the opposite. My body let me down. I feel weak. I feel I don't know my body. I feel distant” (#3611)

#### Self-esteem and sexual esteem

While YGCS seem to develop a “new appreciation for their body” (#3711) post treatment, many described feeling “less sexually attractive” (#3469) or different, “The biggest thing for me was the menopause and not feeling sexy. I just don’t feel like myself” (#7987). Besides menopause, one individual explained how treatment impacted self-esteem and sexual esteem, “hormones, a sense of dullness, vulvar and vaginal discomfort… having that constant reminder is pretty unsexy. It’s not going to make you feel great about yourself” (#6260).

### Theme 3: fear

Fear was expressed in two ways. First, cancer-related fears, “I worry about, is it going to come back or am I going to make someone sick. It never really leaves the back of your mind” (#9547). Second, sex-related fears and the resulting impact on sexual desire and partner relationships, “My sex drive is almost non-existent because I'm worried about sex. I'm worried that it'll hurt… Our sex life nearly broke us up” (#5137). Sexual fears also extended to the (perceived) partner’s fear of causing harm, “I think he was quite afraid to come near me and quite afraid to hurt me” (#7987).

### Theme 4: loss

Loss comes in many forms through a gynaecological cancer journey and can be accompanied by feelings of shock, fear, sadness, anxiety, anger, and acceptance. Table 3 shows ways in which YGCS may experience loss through diagnosis, treatment, and recovery. In this context, YGCS morn aspects of their pre-cancer life or the life they thought they were going to have. Loss appeared to be aggravated by comparing one’s life to someone else’s or to their own pre-cancer life.

**Table 3:** Types of loss

Subtheme	Illustrative quote
Pre-cancer body	“It doesn’t get wet the way it used to. Your body is like on a different planet”. (#2967)
Pre-cancer life	“I’m mentally struggling with the grief, the loss, of my life before cancer.” (#3711)
Identity	“I loss a sense of being a woman. I really miss having periods”. (#5137)
Youth	“I'm young and going through menopause that was really hard for me to digest… a young woman dealing with older women's issues.” (#7987)
Fertility	“Part of that role as a woman to carry and procreate isn’t there anymore. I feel let down because I can’t fulfil that role or that want in my life.” (#1634)
Sex life	“We don't have a sexual life anymore at all. You mourn the loss of your sex life. It’s like everything to do with your sexuality gets put in a coffin.” (#2698)

### Theme 5: shame

Shame was expressed in two ways. First, body shame, “I feel a little self-cautious in myself, a bit gross. It’s really that guilt and that shame for me.” (#9547) and “Physically, I felt unconformable, I’ve got a big scar down the middle of my belly. I had no hair” (#4845). Second, sexual shame, “I’ve always felt that was my fault. Everything was my fault in the bedroom if it wasn’t working” (#5166). Bleeding during sex was also embarrassing even in long-term committed relationships, “I bled quite a bit. I was embarrassed” (#7987).

Additionally, stigma can feed into shame. Cervical cancer survivors reported that the “HPV virus and sexual stigma attached to it… it’s not helping women to have sexual quality of life” (#5137). For example, “A male doctor spoke down to me. You've got that sense of, ‘You've got an STD, you should have been more careful’ He really stigmatised that. It’s embarrassing.” (#9547) The need for change through “spreading awareness and breaking down the stigma” (#5453) extends to female sexuality, “It’s very much ingrained in us, particularly as females, not to talk, or be seen as being demanding or overly sexual. Cultural change needs to happen in that, females have needs and desires sexually as well” (#3611).

### Theme 6: trauma

The trauma of cancer diagnosis and treatment impacts SQoL. Phrases such as “We don’t have a sexual life at all. I really felt sort of violated in a way” (#2698) and “the trauma that came with the treatment… people constantly in that space, monitoring it” (#1634) were used to describe medical trauma. Lastly, the cancer experience can lead to post-traumatic stress and a sense of losing control over one’s life and body.

It’s so hard. I did see a psychologist but it's PTSD. You develop it because you want to control certain aspects of your life and cancer takes that away from you. The biggest thing is, I developed a massive disorder with eating and exercise. It took four years to get on top of it all. (#5453).

### Theme 7: communication

YGCS expressed needing communication and support to cope with the aftermath of gynaecological cancer that hinders SQoL.

#### Communicating sexual needs

YGCS experienced anxiety and frustration related to the lack of available information and support, “This brings a lot of stress to the relationship. I don't think my husband knew what to do” (#1634). Conversely, talking about sex could be harder when feeling guilty or sexually inadequate because of low sexual drive or activity, “As we’ve become closer emotionally and physically, that's probably become less taboo. Probably, I don’t feel quite so guilty talking about it, because I feel like I’m participating as much as he would want it to be” (#4845). Finally, participants felt supported and not pressured into having sex, “I’m definitely supported. He is respectful and supportive. But I suppose it’s me putting on the brakes, trying to figure out what I want, or do I want” (#3611).

#### Patient-doctor communication

Nurses were seen as easy to talk to when it comes to sexual health, “Before treatment began, that was definitely an abrupt conversation with my radiologist. His bedside manner was fairly poor. It was probably best that his nurses had those conversations. They did talk about the shortening, the scarring, the use of dilators, and that some women experience a lack of sex drive” (#1634).

## Discussion

Our interviews indicated that SQoL matters to YGCS, and better communication and support is needed to facilitate adjustment to life after a cancer diagnosis. First, our findings confirmed that single and partnered women experienced high psychosexual distress and the impact of GC diagnosis and treatment is long-lasting. Although some participants commented on the clinical competency of their doctor or medical team, many YGCS claimed they lack the information and the tools needed to manage the physical side effects of treatment, the psychological trauma of cancer and treatment, and the resulting impact on their everyday life and intimate relationships. This lack of communication and support clearly exacerbates feelings of fear, loss, and shame for YGCS. Moreover, confidence and body image concerns are difficult challenges to overcome post GC [31], especially for younger survivors facing early menopause [32].

Second, our research sustained that a supportive partner is a key factor of SQoL for YGCS and may act as a buffer towards better sexual health and QoL [13, 31]. Although cancer treatment can significantly impact physical intimacy, open communication, emotional intimacy, and a willingness to explore new ways of being intimate together may lead to greater relationship satisfaction and SQoL. Consequently, involving partners in treatment education, care planning, providing couple counselling, dispelling myths about sexuality and cancer, offering information that normalises a range of sexual practices, and providing training in partner communication may facilitate mutual understanding, communication and support [10, 33–36]. Healthcare providers should also consider the needs of single women [37, 38] and YGCS in same-sex relationships.

Third, although older GCS have reported shyness in discussing sexual concerns with their medical teams [10], their younger counterparts want to understand how cancer treatment may impact their sexuality and the help that is available to protect and improve SQoL. Our findings aligned with Abbott-Anderson, Young and Eggenberger [31] where participants in our study indicated that they want healthcare providers to initiate those conversations. The need to offer more practical and reassuring information about sexuality to both patients and partners has also been established elsewhere [10, 39]. Hindering factors to patient-doctor communication were described by our participants as the doctor’s bluntness, lack of time, and focus on survival. Other studies reported on the importance of training medical professionals, providing referrals, and the greater involvement of nurses and interdisciplinary care teams to reduce the burden of treatment and medical trauma [5, 17, 40]. Moreover, our study indicates that YGCS experience stigmatisation around female sexuality and HPV. Healthcare providers could play a role in normalising those conversations to reduce stigma and shame [32], for example, using the widely recommended PLISSIT model (Permission, Limited Information, Specific Suggestions, and Intensive Therapy) as a framework to discuss sexuality [31, 40, 41].

### Strengths and limitations

The strengths of this study included the use of an interview guide, which was grounded in evidence and pilot tested. Participants’ data offered a good representation of women living with cervical and ovarian cancers across Australia, in terms of FIGO stages, cancer treatment, age, relationship status, and time since diagnosis. Furthermore, efforts were taken to ensure quotes were presented from across the data set. Finally, the qualitative data was complemented by an assessment of participants’ characteristics and psychosexual distress using a standardised tool (FSDS-R). One limitation was that other types of GC (vaginal, vulvar, uterine) were not represented in the study. Given the relatively small sample size (*n* = 15) caution should be used when generalising findings to populations outside of Australia or to individuals who do not have access to the internet.

## Conclusion

This study found that YGCS experienced high psychosexual distress, and that more support is needed to reduce the impact of treatment on SQoL for both single and partnered women. Findings revealed that the long-lasting impact of gynaecological cancer diagnosis and treatment goes beyond sexual dysfunction and interferes with partner relationships, psychosexual wellbeing, and quality of life. Healthcare providers have a vital role to play in supporting YGCS and their partners across the care continuum, notably by initiating and normalising conversations on the impact of treatment on SQoL and providing avenues for support for patients and partners. A better understanding of the lived experiences of YGCS can help healthcare providers adopt a holistic, patient-centric, and multidisciplinary approach to protect and improve SQoL.

## Supplementary Information

Below is the link to the electronic supplementary material.

Supplementary file1 (PDF 115 kb)

Supplementary file2 (PDF 83 kb)

## Data Availability

The datasets generated and analysed during the current study are not publicly available.

## Code availability

N/A.
