# Supplementary material for: Sexual quality of life in young gynaecological cancer survivors: a qualitative study
Source: Qual Life Res. 2023 Mar 22;32(7):2107–15. doi: 10.1007/s11136-023-03386-1 (PMC10241684; doi:10.1007/s11136-023-03386-1)
Supplement: Supplementary file 1 — Supplementary file1 (PDF 115 kb) [file 11136_2023_3386_MOESM1_ESM.pdf]

The Female Sexual Distress Scale-Revised (FSDS-R; revised 2005): Screening Questionnaire for Measuring Sexually Related Personal Distress in Women With Female Sexual Dysfunction (FSD)

Name: **Date:**

Below is a list of feelings and problems that women sometimes have concerning their sexuality. Please read each item carefully, and circle the number that best describes HOW OFTEN THAT PROBLEM HAS BOTHERED YOU OR CAUSED YOU DISTRESS DURING THE PAST 30 DAYS INCLUDING TODAY. Circle only one number for each item, and take care not to skip any items. If you change your mind, erase your first circle carefully. Read the example before beginning, and if you have any questions please ask about them.

Example: How often did you feel: **Personal responsibility for your sexual problems.**

NEVER	RARELY	OCCASIONALLY	FREQUENTLY	ALWAYS
0	1	2	3	4

How often did you feel

1. Distressed about your sex life	0	1	2	3	4
2. Unhappy about your sexual relationship	0	1	2	3	4
3. Guilty about sexual difficulties	0	1	2	3	4
4. Frustrated by your sexual problems	0	1	2	3	4
5. Stressed about sex	0	1	2	3	4
6. Inferior because of sexual problems	0	1	2	3	4
7. Worried about sex	0	1	2	3	4
8. Sexually inadequate	0	1	2	3	4
9. Regrets about your sexuality	0	1	2	3	4
10. Embarrassed about sexual problems	0	1	2	3	4
11. Dissatisfied with your sex life	0	1	2	3	4
12. Angry about your sex life	0	1	2	3	4
13. Bothered by low sexual desire	0	1	2	3	4

A score of ≥11 effectively discriminates between women with FSD and no FSD.*

Total

Copyright ©2000 by American Foundation for Urological Disease Inc.

* DeRogatis L, et al. *J Sex Med*. 2008;5:357-364.
