# Supplementary material for: Sexual quality of life in young gynaecological cancer survivors: a qualitative study
Source: Qual Life Res. 2023 Mar 22;32(7):2107–15. doi: 10.1007/s11136-023-03386-1 (PMC10241684; doi:10.1007/s11136-023-03386-1)
Supplement: Supplementary file 2 — Supplementary file2 (PDF 83 kb) [file 11136_2023_3386_MOESM2_ESM.pdf]

Sexual quality of life in young gynaecological cancer survivors: A qualitative study

## Interview Guide

### Theme 1: The impact of cancer and its treatment on SQoL

Thank you for taking part in this research. My goal is to create a program that will help women protect and improve their sexual quality of life throughout their cancer journey. You have the option of not answering a question if it makes you feel uncomfortable. My first question is on the impact of cancer and its treatment on sexuality and quality of life. Would it be ok if I ask you some questions about this topic?

1. In what way has cancer and its treatment affected you and your everyday life?
2. How has your diagnosis and treatment affected your sexual life?
3. Is there anything that you feel was (or would have been) helpful in managing the impact of cancer and its treatment on your sexuality? *(Probe – e.g. personal strategies, previous experiences, resources, support, or information).*

### Theme 2: Relationship satisfaction & emotional support

My next question is about relationships and emotional support. Would it be ok if I ask you some questions about this topic?

1. Can you tell me about the place sexuality has in your life or relationship?
2. Have you felt supported as a <relationship status> when it comes to your sexual needs?

*If partnered*

3. Do you find it easy to talk to your partner about sex? What makes you say that?
4. Is there anything that you feel was (or would have been) helpful in strengthening the physical or emotional intimacy with your partner? (Probe – e.g., personal strategies, previous experiences, resources, support, or information.)

## Theme 3: The role of the healthcare system in SQoL

My next question is about the role of the healthcare system in relation to sexuality. Would it be ok if I ask you some questions about this topic?

1. Has anyone spoken to you about the impact of cancer and its treatment on your sexuality – before or after treatment? (Probe: Is there anything you wish you knew or had been told?)
2. Have you been able to discuss your questions or concerns about sexuality with your doctor (or someone from your medical team)? What concerns, if any, was your doctor unable to help you with?
3. In your experience, how could we help improve patient-doctor communication?
4. Is there anything else you would like to add on the role of the healthcare system when it comes to sexuality – before, during or after treatment?

## Theme 4: The adaptation to a new normal

My final questions are about social support and the adaptation to a new normal after cancer. Would it be ok if I ask you some questions about this topic?

1. Have you noticed any changes to how you feel about your body since treatment? (Probe: Do you think differently of yourself or your body since cancer? If so, in what ways?)
2. Have you noticed any changes to how you see yourself sexually since your diagnosis? (Probe: Have you noticed any changes on how attractive you feel?)
3. Do you feel self-pleasure is important for women recovering from cancer? (Probe: What role does self-pleasure play in the rediscovery of sexuality?)
4. Have you discussed having <cancer type> with others? Can you comment on whether this was helpful to you?
5. Some women have expressed having difficulty in accepting help following their cancer diagnosis and treatment, what would you advise a woman who is reluctant to accept help?
6. Has cancer had any positive effects for you?

7. What does sexual quality of life mean to you?
8. This concludes our interview. Is there anything else you wish to add that may help other YGCS?

Thank you for taking the time to share your experience and insights to help other women living with cancer.
